# Supplementary material for: A fault-intrusion-tolerant system and deadline-aware algorithm for scheduling scientific workflow in the cloud
Source: PeerJ Comput Sci. 2021 Nov 2;7:e747. doi: 10.7717/peerj-cs.747 (PMC8576554; doi:10.7717/peerj-cs.747)
Supplement: Supplemental Information 6 [file peerj-cs-07-747-s006.zip › Myproject1.1/docs/org/cloudbus/cloudsim/class-use/CloudletScheduler.html]

Uses of Class org.cloudbus.cloudsim.CloudletScheduler


JavaScript is disabled on your browser.


- Overview
- Package
- Class
- Use
- Tree
- Deprecated
- Index
- Help

- Prev
- Next

- Frames
- No Frames

- All Classes

## Uses of Class org.cloudbus.cloudsim.CloudletScheduler

- Packages that use CloudletScheduler

  | Package | Description |
  | --- | --- |
  |  |  |
  | --- | --- |
  | org.cloudbus.cloudsim |  |
  | org.cloudbus.cloudsim.network.datacenter |  |
  | org.cloudbus.cloudsim.power |  |
  | org.workflowsim |  |
- - ### Uses of CloudletScheduler in org.cloudbus.cloudsim

    Subclasses of CloudletScheduler in org.cloudbus.cloudsim

    | Modifier and Type | Class and Description |
    | --- | --- |
    |  |  |
    | --- | --- |
    | `class` | `CloudletSchedulerDynamicWorkload` CloudletSchedulerDynamicWorkload implements a policy of scheduling performed by a virtual machine assuming that there is just one cloudlet which is working as an online service. |
    | `class` | `CloudletSchedulerSpaceShared` CloudletSchedulerSpaceShared implements a policy of scheduling performed by a virtual machine. |
    | `class` | `CloudletSchedulerTimeShared` CloudletSchedulerTimeShared implements a policy of scheduling performed by a virtual machine. |

    Methods in org.cloudbus.cloudsim that return CloudletScheduler

    | Modifier and Type | Method and Description |
    | --- | --- |
    |  |  |
    | --- | --- |
    | `CloudletScheduler` | Vm.`getCloudletScheduler()` Gets the vm scheduler. |

    Methods in org.cloudbus.cloudsim with parameters of type CloudletScheduler

    | Modifier and Type | Method and Description |
    | --- | --- |
    |  |  |
    | --- | --- |
    | `protected void` | Vm.`setCloudletScheduler(CloudletScheduler cloudletScheduler)` Sets the vm scheduler. |

    Constructors in org.cloudbus.cloudsim with parameters of type CloudletScheduler

    Constructor and Description

    `Vm(int id,
    int userId,
    double mips,
    int numberOfPes,
    int ram,
    long bw,
    long size,
    java.lang.String vmm,
    CloudletScheduler cloudletScheduler)`

    Creates a new VMCharacteristics object.
  - ### Uses of CloudletScheduler in org.cloudbus.cloudsim.network.datacenter

    Subclasses of CloudletScheduler in org.cloudbus.cloudsim.network.datacenter

    | Modifier and Type | Class and Description |
    | --- | --- |
    |  |  |
    | --- | --- |
    | `class` | `NetworkCloudletSpaceSharedScheduler` CloudletSchedulerSpaceShared implements a policy of scheduling performed by a virtual machine. |

    Constructors in org.cloudbus.cloudsim.network.datacenter with parameters of type CloudletScheduler

    Constructor and Description

    `NetworkVm(int id,
    int userId,
    double mips,
    int pesNumber,
    int ram,
    long bw,
    long size,
    java.lang.String vmm,
    CloudletScheduler cloudletScheduler)`
  - ### Uses of CloudletScheduler in org.cloudbus.cloudsim.power

    Constructors in org.cloudbus.cloudsim.power with parameters of type CloudletScheduler

    Constructor and Description

    `PowerVm(int id,
    int userId,
    double mips,
    int pesNumber,
    int ram,
    long bw,
    long size,
    int priority,
    java.lang.String vmm,
    CloudletScheduler cloudletScheduler,
    double schedulingInterval)`

    Instantiates a new power vm.
  - ### Uses of CloudletScheduler in org.workflowsim

    Constructors in org.workflowsim with parameters of type CloudletScheduler

    Constructor and Description

    `CondorVM(int id,
    int userId,
    double mips,
    int numberOfPes,
    int ram,
    long bw,
    long size,
    java.lang.String vmm,
    CloudletScheduler cloudletScheduler)`

    Creates a new CondorVM object.

- Overview
- Package
- Class
- Use
- Tree
- Deprecated
- Index
- Help

- Prev
- Next

- Frames
- No Frames

- All Classes
